# Oral health-related quality of life in complete denture wearers depending on their socio-demographic background, prosthetic-related factors and clinical condition

**DOI:** 10.4317/medoral.18648

**Published:** 2013-03-25

**Authors:** Carmen Perea, María J. Suárez-García, Jaime Del Río, Daniel Torres-Lagares, Javier Montero, Raquel Castillo-Oyagüe

**Affiliations:** 1 D.D.S. Researcher. Department of Buccofacial Prostheses, Faculty of Odontology. Complutense University of Madrid (UCM), Pza. Ramón y Cajal, s/n, 28040, Madrid, Spain; 2 M.D., D.D.S., Ph.D. Professor. Department of Buccofacial Prostheses, Faculty of Odontology. Complutense University of Madrid (UCM), Pza. Ramón y Cajal, s/n, 28040, Madrid, Spain; 3 M.D., D.D.S., Ph.D. Cathedratic Professor. Department of Buccofacial Prostheses, Faculty of Odontology. Complutense University of Madrid (UCM), Pza. Ramón y Cajal, s/n, 28040, Madrid, Spain; 4 D.D.S., Ph.D.Tenured Professor. Faculty of Dentistry. University of Seville (US), C/ Avicena, s/n, 41009, Seville, Spain; 5 D.D.S., Ph.D. Tenured Professor, Department of Surgery, Faculty of Medicine. University of Salamanca (USAL), Campus Miguel de Unamuno, s/n, 37007, Salamanca, Spain; 6 D.D.S., Ph.D. Associate Professor. Department of Buccofacial Prostheses, Faculty of Odontology. Complutense University of Madrid (UCM), Pza. Ramón y Cajal, s/n, 28040, Madrid, Spain

## Abstract

Objectives: To investigate the differences in impact on oral health-related quality of life (OHRQoL) among complete denture wearers depending on their socio-demographic characteristics, prosthetic-related factors and oral status. Study Design: 51 patients aged 50-90 years treated, from 2005 to 2010, with at least one complete denture at the Department of Buccofacial Prostheses of the Complutense University (Madrid) were enrolled in this cross-sectional study. All of the participants answered the Oral Health Impact Profile (OHIP-14sp) questionnaire. The additive scoring method was used. The prevalence of impacts was calculated by using the occasional threshold (OHIP-14sp score≥2). Socio-demographic and prosthetic-related variables were gathered. Patients underwent clinical examination to assess their oral condition. Descriptive probes and Chi-Square tests were run (p≤0.05). Results: The predominant participants’ profile was that of a man with a mean age of 69 years wearing complete dentures in both the maxilla and the mandible. The prevalence of impact was 23.5%, showing an average score of 19±9.8. The most affected domains were “functional limitation” and “physical pain”, followed by “physical disability”. Minor impacts were recorded for the psychological and social subscales (“psychological discomfort”, “psychological disability”, “social disability” and “handicap”). The prosthesis’ location significantly influenced the overall patient satisfaction, the lower dentures being the less comfortable. Having a complete removable denture as antagonist significantly hampered the patient satisfaction. Patients without prosthetic stomatitis and those who need repairing or changing their prostheses, recorded significantly higher OHIP-14sp total scores. Conclusions: The use of conventional complete dentures brings negative impacts in the OHRQoL of elderly patients, mainly in case of lower prostheses that required reparation or substitution, with a removable total denture as antagonist. The prosthetic stomatitis in this study was always associated to other severe illness, which may have influenced the self-perceived discomfort with the prostheses, as those patients were daily medicated with painkillers.

** Key words:**Oral Health Impact Profile (OHIP), oral health-related quality of life (OHRQoL), patient satisfaction, complete denture, elderly patients.

## Introduction

Notwithstanding the long-term success of implant-based restorations, the world population growth rates along with the extended life expectancy may lead to an increasing demand for conventional removable dentures. Moreover, this treatment modality allows avoiding surgical risks, difficulties and costs associated with implant prostheses (1). The complete edentation influences the well-being and life satisfaction of individuals (2). Also the use of conventional full dentures could have adverse effects on their oral health-related quality of life (OHRQoL). Although several instruments have been developed to assess the functional, social and psychological outcomes of oral disorders by using a methodological approach (3,4), there is no specific application to assess the impact of conventional dentures in OHRQoL. Nonetheless, using a generic health status scale may enable to compare more easily the results (5), which will prove the removable prostheses’ real effectiveness in restoring the oral function taking also into account the patients’ subjectivity when they express their feelings.

In 1994, Slade and Spencer (6) introduced the Oral Health Impact Profile (OHIP-49) questionnaire, containing 49 questions that capture seven conceptually formulated dimensions (“functional limitation”, “physical pain”, “psychological discomfort”, “physical disability”, “social disability” and “handicap”), based on the Locker’s theoretical model of oral health (7). Despite its wide acceptance, proven reliability and strong validity, the large number of items included in this instrument may limit its use in clinical trials, clinical practice and surveys (8). When choosing measurement scales to evaluate the OHRQoL in the elderly, short questionnaires seem to have more advantages (9). Accordingly, in 1997, Slade (10) published a short form with the same dimensions (OHIP-14) that confirmed comparable results to those achieved with the original version of the OHIP. Furthermore, as any study based on questionnaires must take into account the socio-demographic characteristics of the population, translating and validating these assessment tools in different languages are required to consider the possible influence of socio-cultural factors on the self-perception of oral health (11).

This is the first study focused on the overall satisfaction of edentulous patients treated with conventional dentures after the OHIP-14 scale was validated for Spanish inhabitants (12). The information obtained may be useful in predicting with some caution the impact of this type of rehabilitation in the quality of life of patients from Spain and other countries that have related socio-demographic, cultural and clinical features. Therefore, the purpose of this investigation is to evaluate the differences in impact on OHRQoL among elderly complete denture bearers, using the Oral Health Impact Profile (OHIP-14) indicator.

## Material and Methods

-Study protocol

The reference population was 118 patients aged 50-90 years treated, between 2005 and 2010, with at least one conventional complete denture at the Department of Buccofacial Prostheses of the Complutense University of Madrid. The exclusion criteria were: cognitive impairment, motility disorders and serious illness. 62 patients were invited by telephone to take part in the study. Each of the 51 final volunteers was scheduled for an appointment that consisted of an interview and a clinical examination free of charge. The Approval Ethics Committee (C.E.I.C., San Carlos University Hospital, Madrid. C.P. - C.I. 12/240-E) was obtained, as the study was conducted following the ethical principles of medical investigation involving human subjects under the Helsinki Declaration of the World Medical Association (http://www.wma.net) and the Spanish Law 14/2007 of July 3rd for Biomedical Research (http://www.boe.es). All of the participants were informed of the aims and procedures of the study. The patients’ approved written consent was requested and confidentiality was maintained.

First of all, subjects completed a questionnaire supplying information on their socio-demographic background (age, gender, marital status, education level) and behavioral factors (smoking and drinking habits) (Group 1 of study variables).

Afterwards, the assessment of the technical conditions of the prostheses was performed by a single researcher. The following denture-related data were registered: date of installation of the prosthesis, location and type of opposite prosthetic treatment (Group 2 of study variables).

The diagnosis of the patients’ oral health status was carried out by the same clinician. Time of edentulism, mobility of the masticatory mucosa, type of alveolar ridge, presence of prosthetic stomatitis, dry mouth sensation and need of treatment were recorded (Group 3 of study variables).

Four categories were established to classify the type of residual ridge that supported the complete removable prosthesis: Type 1: high wide ridge; type 2: high narrow ridge; type 3: low wide ridge; and type 4: low narrow ridge. Chronic inflammation of the denture-bearing mucosa, which was detected by direct visual inspection, was considered as “prosthetic stomatitis” (13). The requirement of treatment could involve medical management and control of oral lesions and/or repairing or changing the prostheses.

Participants that had worn previous complete dentures were asked about changes in their aesthetic appearance and chewing ability (better, worse, or equal) since they began using the prostheses analyzed in the present study.

Finally, the OHRQoL was assessed using the OHIP-14sp (Spanish validated version of the OHIP-14 generic indicator) (12). A trained examiner applied the questionnaire in the form of a face-to-face interview. The volunteers answered in terms of frequency the appearance of 14 situations of impact conceptually divided into seven domains or dimensions. Each response was codified with one of the following options of a five-point Likert scale: “never” (score 0), “hardly ever” (score 1), “occasionally” (score 2), “fairly often” (score 3) and “very often” (score 4). To minimize the response bias a 1-month recall period was considered. The OHIP-14 outcome variable may range from 0 to 56 points, such that the lower the total score was, the minor impact on OHRQoL was and, thus, the greater the satisfaction and well-being of the patient were.

-Data analysis 

All data analyses were made by using the Statistical Package for the Social Sciences (SPSS/PC+ v.19, Inc.; Chicago, IL, USA). In order to calculate not only the total OHIP-14sp output, but also the score per dimension, the additive method (OHIP-ADD) was used by adding (a) the scores recorded for the 14 items of the test and (b) the scores obtained for the two questions of each domain. The prevalence of impact was calculated by using the occasional threshold (score ≥ 2). Thus, a subject was considered with impact, if at least one item of the OHIP-14 was reported in and occasional or more frequently manner (score ≥ 2).

Descriptive statistics were calculated for all of the socio-demographic, prosthetic and clinical variables. Due to the fact that the Kolmogorov-Smirnov test confirmed that the OHIP-14sp outcome did not follow a normal distribution, the prevalence of impact on OHRQoL was compared between groups using the Chi Square test. A p-value < 0.05 was considered as statistically significant.

## Results

-Analysis of socio-demographic, prosthetic and clinical variables

From the reference population (n = 118), 67 patients (56.78%) were excluded because of cognitive impairment (n = 1) or contact impossibility due to changes in their phone number and/or address details (n = 55 patients). A total of 11 patients refused to participate (rejection rate = 9.32%). The final pool of patients comprised 51 individuals. The most relevant statistical outcomes are shown in tables  Table 1, Table 2, Table 3. Relating to the socio-demographic and behavioral variables (Group 1), the study sample was drawn mainly from men (70.6%), with a predominant age range of 65-74 years (43.1%), married (76.5%) and with a basic level of education (78.4%). Moreover, most participants were non-smokers (82.3%) and non- drinkers (74.5%) ( Table 2).

**Table 1:**
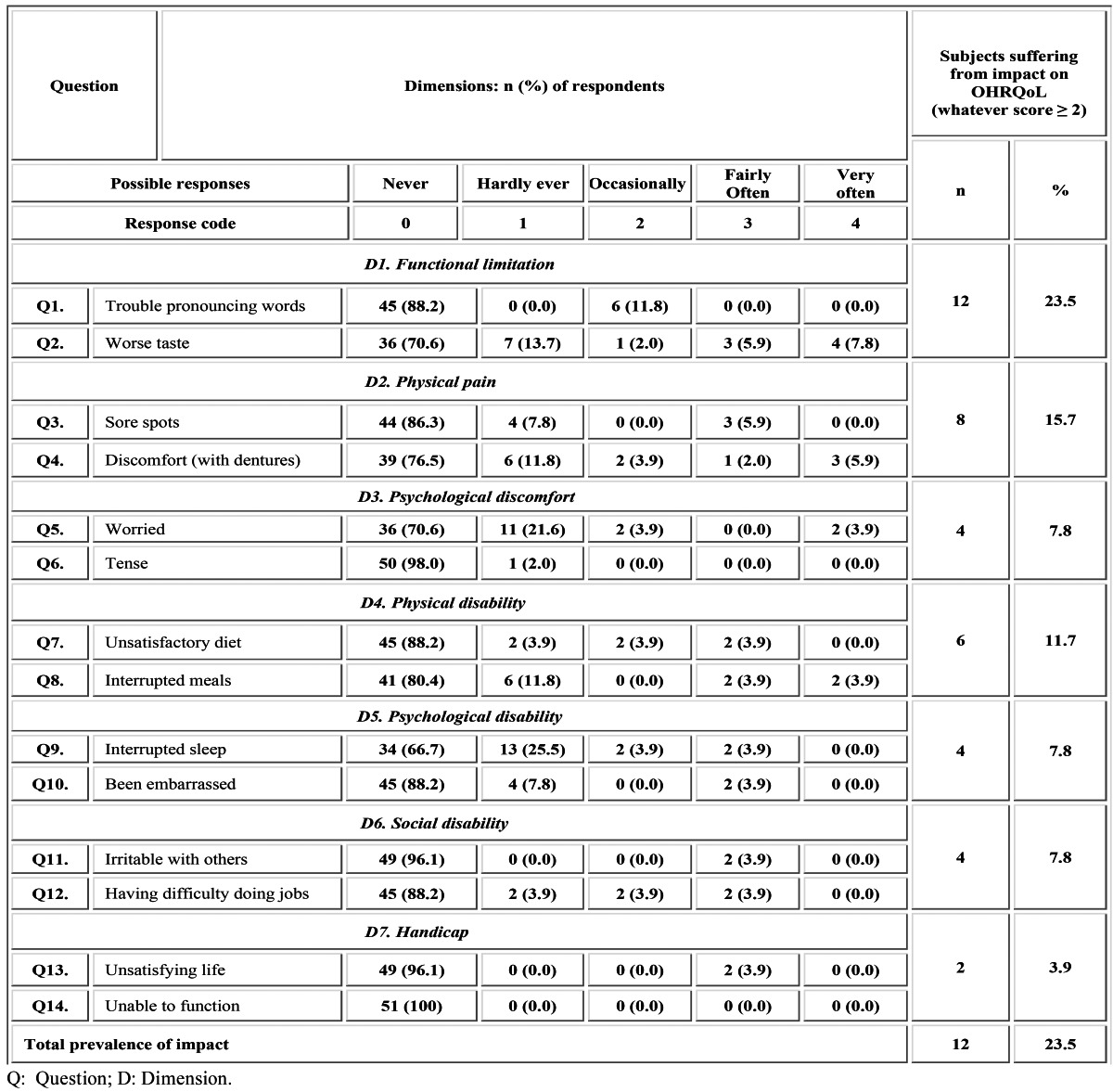
Prevalence of impact on OHRQoL according to the domains and questions of the OHIP-14sp scale.

**Table 2:**
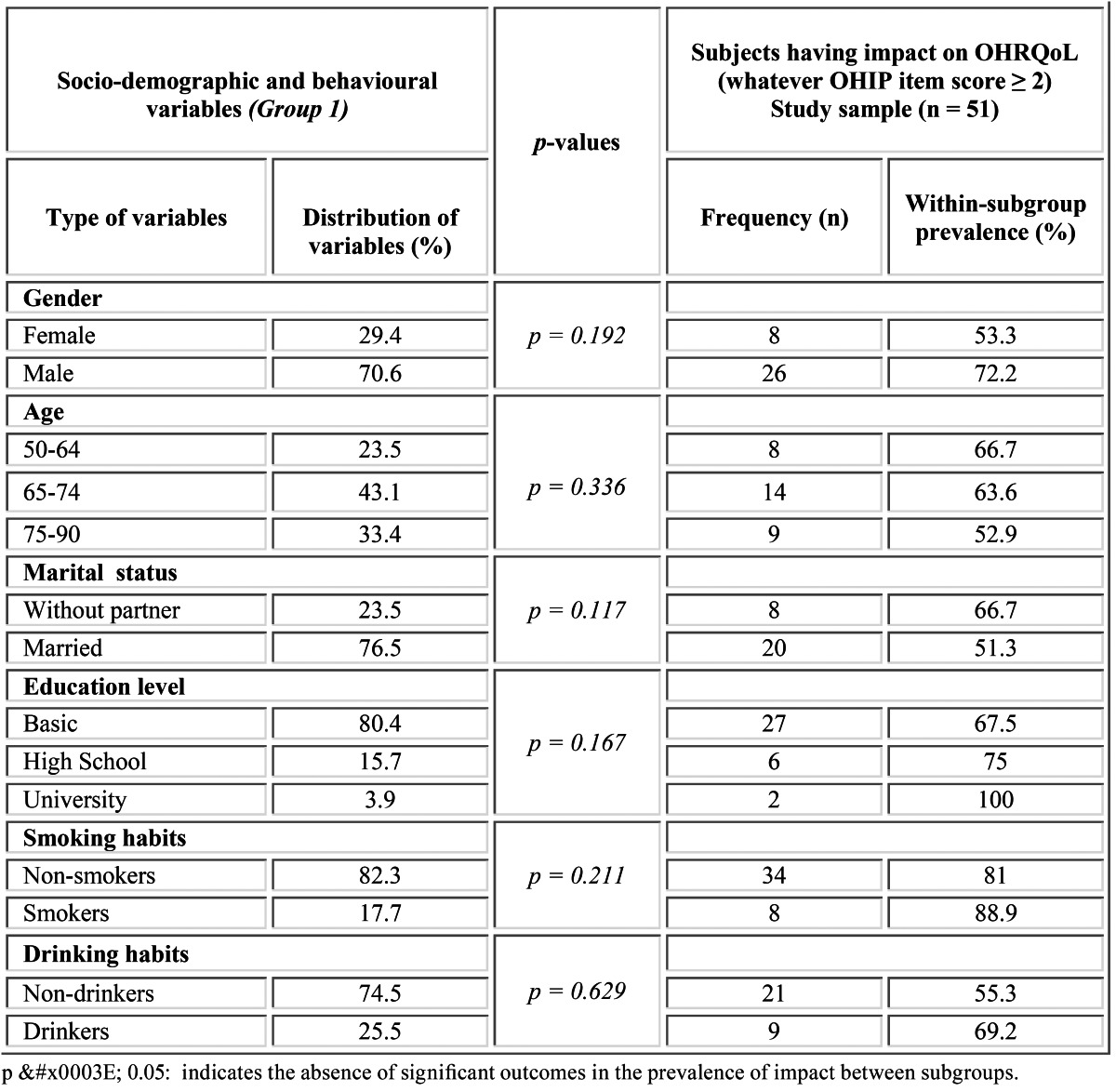
Prevalence of impact on OHRQoL as regards the socio-demographic and behavioural variables.

**Table 3:**
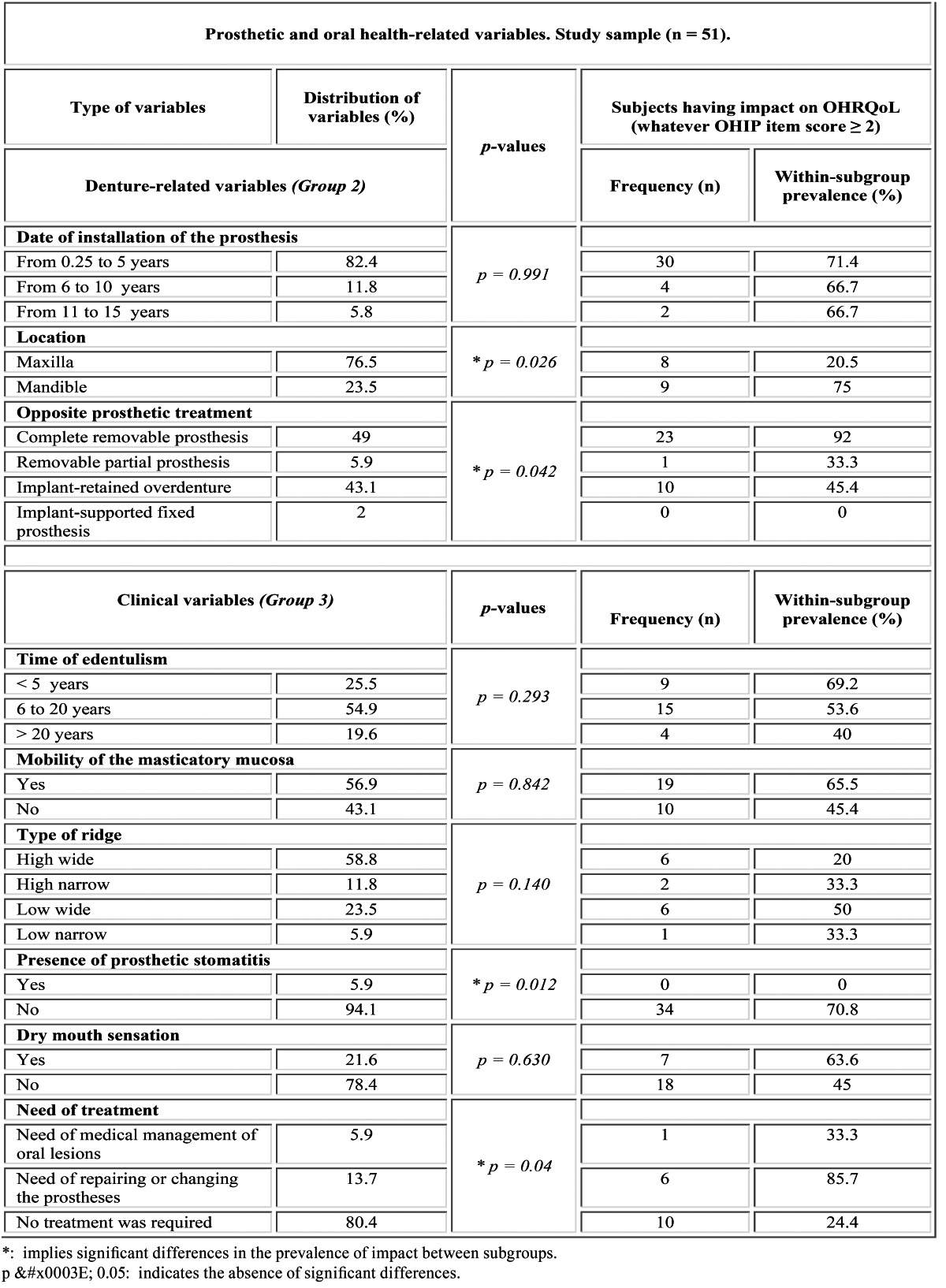
Prevalence of impact on OHRQoL as regards the oral health-related variables.

Concerning the denture-related factors (Group 2), 82.4 % of the patients had worn their prostheses for a period of less than five years and 76.5% of the volunteers wore their complete dentures in the maxilla. The antagonist prosthetic treatment was a complete removable prosthesis (49%), an implant-retained overdenture (43.1%), a removable partial prosthesis (5.9%) or an implant-supported fixed denture (2%) ( Table 3).

**Figure 1:**
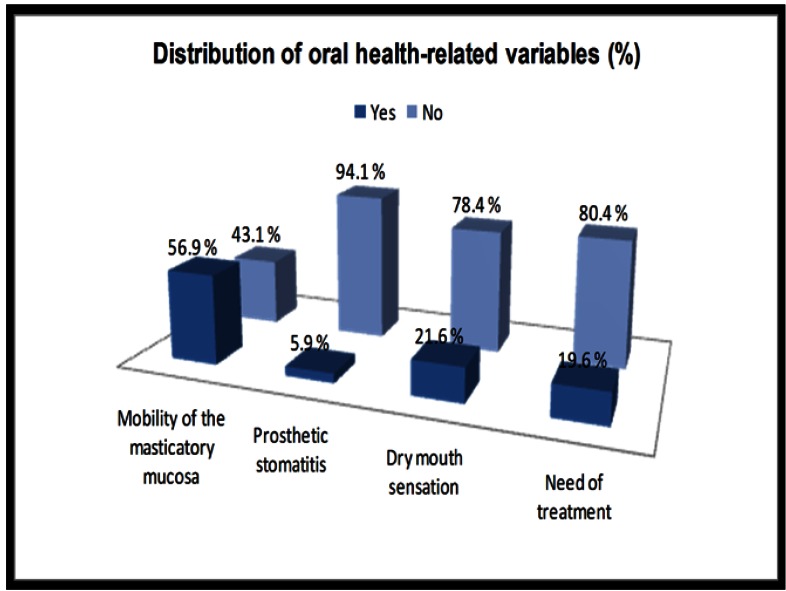
Distribution of oral health-related variables in the study sample (%).

Regarding the clinical variables (Group 3), the mean time of edentulism was 15.5 ± 13.1 years. The masticatory mucosa presented mobility in 56.9% of cases. Sorted in descending order of frequency, 58.8% of the patients had a high wide ridge supporting the tested prostheses (type 1), 23.5% had a low wide ridge (type 3), 11.8% had a high narrow ridge (type 2) and 5.9% had a low narrow ridge (type 4). Prosthetic stomatitis was found in 5.9% of patients, whereas 21.6% of the participants reported a dry mouth sensation. 80.4% of the patients did not require any prosthetic-related treatment. 13.7% of participants needed repairing or changing their prostheses, whereas 5.9% of the volunteers required medical management of their oral lesions ( Table 3, Fig. 1).

Prevalence of impacts (OHIP-14sp) 

No questionnaires had to be eliminated from the study because all of the items were properly filled out in each case.  Table 1 shows the most prevalently affected OHIP subscales. 23.5% of the participants reported at least one impact in an occasional or more frequently manner during the last month ( Table 1). The average OHIP-14sp total score was 19 ± 9.8.

In view of the occasional threshold (score ≥ 2), the most affected dimensions or domains (D) were “functional limitation” (23.5% of prevalence) and “physical pain” (15.7%) followed by “physical disability” (11.7%). Minor prevalence was recorded for the psychological and social subscales. Thus, the frequency of “psychological discomfort”, “psychological disability” and “social disability” was 7.8%, whereas the “handicap” dimension resulted in a prevalence of 3.9% ( Table 1).

**Figure 2:**
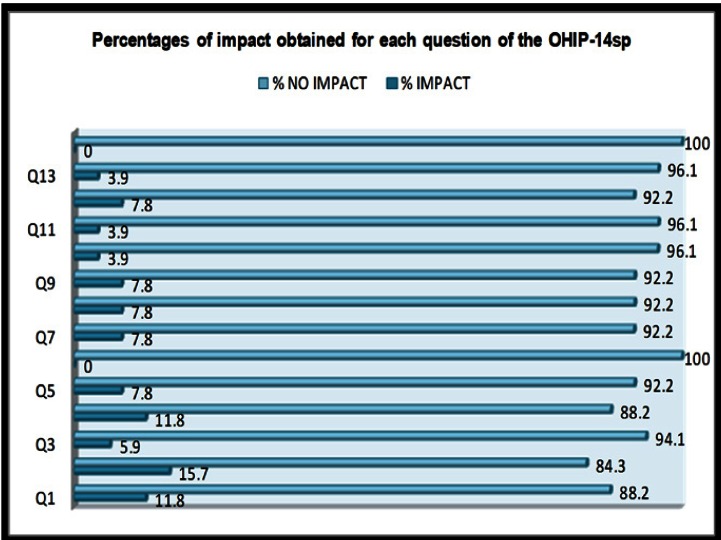
Percentages of impact obtained for each question of the OHIP-14sp. The 14 items of the questionnaire are grouped in the next dimensions/ domains: “functional limitation” (Q1 and Q2); “physical pain” (Q3 and Q4); “psychological discomfort” (Q5 and Q6); “physical disability” (Q7 and Q8); “psychological disability” (Q9 and Q10); “social disability” (Q11 and Q 12); “handicap” (Q13 and Q14).

Detailing the analysis of the OHIP scores obtained per question (Q), all of the participants reported “no impact” for being tense (Q6, D3) or feeling unable to function (Q14, D7). The main problems were found in worse taste (Q2, D1), which occurred occa-sionally or more frequently in 15.7% of cases. Subsequently, trouble pronouncing words (Q1, D1) and discomfort with dentures (Q4, D2) showed a prevalence of 11.8%. Intermediate values were found for feeling worried (Q5, D3), unsatisfactory diet (Q7, D4), interrupted meals (Q8, D4), interrupted sleep (Q9, D5) and experiencing some difficulties doing jobs (Q12, D6); all of them being reported by 7.8% of the patients. Minor prevalence of impact (5.9%) was registered for sore spots (Q3, D2). Finally, being embarrassed (Q10, D5), being irritable with others (Q11, D6) and having an unsatisfying life (Q13, D7), showed a prevalence of 3.9% ( Table 1, Fig. 2).

The following modulating factors resulted in the highest prevalence of impact in quality of life (OHIP-14sp score ≥ 2):

Group 1: males (72.2%), within an age range of 50 to 64 years (66.7%), without partner (66.7%), having university education (100%) and being smokers (88.9%) and drinkers (69.2%) ( Table 2). However, no significant differences were recorded for such socio-demographic and conductual variables.

Group 2: wearing the prosthesis for less than 5 years (71.4%), having a full lower denture (75%) and an opposite complete denture (92%) ( Table 3). The prosthesis’ location significantly influenced the patient overall satisfaction, the lower dentures being the less comfortable (p = 0.026). Furthermore, the “functional limitation” and “physical pain” dimensions showed significantly higher prevalence of impact in patients who wore lower complete dentures (p < 0.01). Significant differences were found depending on the type of opposite prosthetic treatment (p = 0.042), so that opposing complete removable dentures resulted in the lower patient satisfaction (p < 0.05) ( Table 3).

Group 3: being edentulous for less than 5 years (69.2%), having mobility of the masticatory mucosa (65.5%) and low-wide-shaped ridges supporting the denture (50%), absence of prosthetic stomatitis (70.8%), patients reporting dry mouth sensation (63.6%) and needing reparation of their complete prostheses (85.7%).

Significantly lower prevalence of impact was achieved for patients with prosthetic stomatitis (p = 0.012) and for those who required reparation or substitution of their conventional prostheses (p < 0.05) ( Table 3).

Although no significant differences were recorded, participants reporting a dry mouth sensation showed a trend of attaining higher prevalence of impact on the “physical pain” dimension (53%).

In addition, whereas all of the volunteers that had worn previous prostheses (78%) experienced aesthetic improvements since they wore the tested dentures, 64.3% of them noticed positive changes in their chewing ability.

## Discussion

This paper describes the general satisfaction of conventional complete denture wearers on the basis of cross-sectional survey-based data and clinical examination. The sample size was similar to that of related studies on the influence of prosthetic rehabilitations in OHRQoL (14). The results obtained may help predict the possible effect of conventional prostheses in terms of the well-being of future patients. Although one limitation of the research protocol is that the participants were recruited only from a university dental clinic, due to the variability in the gender, age, marital status, level of education and behavioural habits of the volunteers (Fig. 1), our findings might be indicative for patients from other countries having comparable socio-demographic and clinical profiles. Nevertheless, the results of this study should be extrapolated with caution, taking into account that the sample size and the recruitment method may hamper their worldwide application.

The Oral Health Impact Profile generic scale has demonstrated better performance than other questionnaires (15) and higher sensitivity to detect dissatisfaction. It has previously been applied in clinical trials and cross-sectional studies to evaluate the effectiveness of treatments for edentulism (5,16). The use of the OHIP in the present investigation facilitated the comparison of the results. For the same reason, the occasional threshold, which considers as impact responses those scored ≥ 2 (12,17), was chosen. The Spanish validated version of the OHIP-14 (12) was applied in the interview format. Whereas Souza et al. (18) reported no scoring differences regarding the form of administration (self-completed vs. interview), Ekanaye and Perera (2) found lower completion rates and loss of data when the OHIP-14 was self-filled.

A one-month recall period was considered to report impacts instead of a twelve-month recall period that was used in the original source (6). Even though John et al. (19) confirmed that the remind period did not affect the internal consistency of the OHIP, short-term memory is expected to be more accurate to provide a reliable information (2).

The prevalence of impact obtained in this study (23.5%) ( Table 1), which is the percentage of subjects reporting at least one item affected in an occasional or more frequently manner (scored ≥ 2), is less than one third of that previously reported for the Spanish population (12,17). However, it is meaningful that, unlike what happened in such studies, our patients were older and not seeking any treatment. It has been reported that the higher the age, the more frequent the impacts, which has been attributed to the accumulative kind of the oral pathology, such as tooth decay or periodontal disease (17). However, the present investigation was performed on edentulous patients, which may explain to some extent the absence of direct correlation between age and oral impact prevalence. Concerning the OHIP-14sp total average score obtained (19 ± 9.8), Emami et al. (20) suggested that, although mandibular implant-retained overdentures may be more satisfying for edentulous patients than new conventional dentures, the magnitude of the effect still remains to be ascertained. Therefore, there is a need for additional evidence including cost-effectiveness analyses on the impact of mandibular implant overdentures and conventional prostheses.

With regard to the major prevalence of impact, it was found that “functional limitation” (D1) and “physical pain” (D2) were the main causes behind the general patients’ concern, being responsible for worse taste (Q2) followed by trouble pronouncing words (Q1, D1) and unpleasant sensations with dentures (Q4, D2), which were the most common problems included in such dimensions. “Physical disability” (D4) was the third most affected domain, revealing marked diet dissatisfaction (Q7) and interrupted meals (Q8) ( Table 1, Fig. 2). Alteration of taste and fear of losing the denture while eating or talking are consequence of the intrinsic limitations of the complete denture treatment, such as low masticatory performance, compromised retention and stability and coating of palatal minor salivary glands (18). De Oliveira and Frigerio (21) reported that complete denture users could be even more susceptible to malnutrition when compared to implant-retained overdenture wearers.

“Psychological discomfort”, “psychological disability” and “social disability” (D3, D5 and D6, respectively) were less prevalent for the occasional threshold. Complete denture bearers scarcely complained about feeling embarrassed (Q10, D5) or being irritable (Q11, D6) when they wore their prostheses. No participants referred getting nervous with their rehabilitations (Q6, D3). However, the highest incidence of the social domains corresponded to being worried (Q5, D3), having interrupted sleep (Q9, D5) and difficulty doing jobs (Q12, D6) ( Table 1, Fig. 2). This reveals a positive perception for these domains; which agrees with the trend observed by Slade and Spencer (6) when they used the original version of the questionnaire (OHIP-49). Concerning the segmented sleep, dentists generally recommend removal of dentures during the night, since constant wearing can increase the risk of irritations and infections. However, around 10% of people with obstructive sleep apnoea (OSA) who wear complete dentures may experience increased breathing difficulties if they sleep with their prostheses out. Recent findings suggest that in patients with OSA, the advantages of removing dentures during sleep should be weighted against the risk of worsening upper airway collapse (22). Therefore, this factor should be further evaluated by monitoring the patients to correlate the presence of OSA with the score obtained in the Q9 (D5) of the OHIP when patients sleep with or without their prostheses.

The “handicap” subscale (D7) disclosed the best overall satisfaction with the existence as a general concept (Q13) and the ability in the development of ordinary life (Q14) when patients used their conventional dentures. Thus, no patients in this research felt unable to function (Q14) ( Table 1, Fig. 2). Additionally to the greater tolerance to disability of mature patients (23), it is likely that subjective, patient-related feelings have been the major deciding factors of satisfaction concerning this domain.

Gender yielded no significant differences in our study, although there seems to be a marked tendency to higher impact and lower satisfaction in men ( Table 2). These data agree with the findings of Slade and Spencer in edentulous patients (6). Some authors stated the independence of this factor on the subjective perception of OHRQoL (17,23) whereas others reported opposite results (5,12). Therefore, the effect and magnitude of this variable should be further assessed. Besides, the age was not a modulating factor of OHRQoL in the present research ( Table 2). This may be justified because of the reduced age range of the sample. Married patients tended to express higher overall satisfaction with their conventional dentures than those without partner, although no significant differences were encountered ( Table 2). This issue requires further validation, as no previous related study analyzed this variable.

People with higher educational level showed a trend toward a higher impact in their quality of life with no significant differences with respect to those having basic education ( Table 2). Such tendency was announced by McGracth and Bedi in the U.K. (23) and Montero et al. in Spain (17). Moreover, a slightly higher percentage of impact was found in smokers and drinkers ( Table 2). Even though no significant differences were recorded, this tendency concurs with the findings of Lin et al. (24), who reported a higher incidence of oral lesions in smoker and drinker patients.

Being edentulous and wearing the denture for less than five years resulted in higher impacts, as it takes time for patients to get used to removable prostheses ( Table 3). Full lower denture wearers confirmed significantly lower overall satisfaction ( Table 3), which may be due to the centrifugal resorption pattern of the mandible that affects the osteomucosal support of the residual bridge (25). Consistently with Fenlon and Sherriff (26), subjects having a plane flange reported less satisfaction and higher impact in OHRQoL. This mainly occurred when the masticatory mucosa was mobile and not keratinized ( Table 3), leading to lower resistance to trauma. Patients often express dissatisfaction with their mandibular prostheses, complaining about retention stability and difficulties with mastication and verbal communication (27). Accordingly, the “functional limitation” and “physical pain” dimensions showed significantly higher levels of impact when the complete dentures were located in the mandible.

Considering the antagonist prosthetic treatment, at one with Hogenius et al. (28), the lowest prevalence of impact in OHRQoL is characteristic of patients wearing implant-supported fixed prostheses, followed by removable partial dentures in the opposite jaw. Intermediate impact values were recorded when the antagonist was an implant-retained overdenture. The highest impact prevalence corresponded to patients wearing complete dentures in both the maxilla and the mandible, showing significant differences with the other subgroups ( Table 3). Awad et al. (16) found that implant-based treatments significantly improved the health-related quality of life outcome when compared with conventional dentures.

Having a dry mouth sensation resulted in higher impact in patients’ quality of life taking into account that saliva plays an im-portant role in retention and comfort of removable prostheses. However, no significant differences were detected in the present study ( Table 3). The dry mouth sensation has been associated with age and pharmacotherapy (29). In our investigation, patients who expressed dry mouth sensation were medicated for thyroid problems, sleepiness, hypertension, Parkinson, epilepsy or pros-tate cancer, among others.

Significant differences were identified depending on the presence of prosthetic stomatitis, so that patients with such disease showed no impact in OHRQoL ( Table 3). In this research, prosthetic stomatitis was always associated to other severe illness, such as cancer. Thus, the self-perception of discomfort with the prostheses may have faded into the background in case of these patients. Moreover, all participants having severe illness in the present study were daily medicated with painkillers, which may reduce the impact of their prostheses in OHRQoL (29). However, the lack of studies correlating the presence of severe illness (resulting in diseases such as prosthetic stomatitis) and pharmacotherapy with the level of impact in OHRQoL makes comparisons difficult. Therefore, this issue should be further evaluated in different and larger populations to redefine this conclusion.

Patients who required repairing or changing their prostheses expressed significantly lower satisfaction  Table 3), as previously reported (14).

In this study, both the self-perceived aesthetic appearance and the chewing ability improved in most patients who had worn other conventional dentures. Such results are related to those obtained by using the OHIP-14sp, as chewing ability is one of the determinants of denture satisfaction best associated with OHRQoL (30).

To summarize, the following may be concluded: (1) Conventional complete dentures bring negative impacts in the OHRQoL of elderly patients, mainly concerning functional limitation and physical pain. (2) Maxillary conventional dentures are more com-fortable than mandibular ones. (3) The overall patient satisfaction as regards OHRQoL is hampered by having a total removable prosthesis as antagonist. (4) The self-perceived discomfort with conventional dentures faded into the background in patients with prosthetic stomatitis, who always suffered from other severe illness in the present study and were daily medicated with painkillers. (5) The requirement of repairing or changing the prostheses resulted in higher impact in OHRQoL.

## References

[1] Walton JN, MacEntee MI (2005). Choosing or refusing oral implants: a prospective study of edentulous volunteers for a clinical trial. Int J Prosthodont.

[2] Ekanayake L, Perera I (2003 ). Validation of a Sinhalese translation of the Oral Health Impact Profile-14 for use with older adults. Gerodontology.

[3] Locker D, Matear D, Stephens M, Lawrence H, Payne B (2001). Comparison of the GOHAI and OHIP-14 as measures of the oral health-related quality of life of the elderly. Community Dent Oral Epidemiol.

[4] Tsakos G, Allen PF, Steele JG, Locker D (2012). Interpreting oral health-related quality of life data. Community Dent Oral Epidemiol.

[5] Preciado A, Del Río J, Suárez-García MJ, Montero J, Lynch CD, Castillo-Oyagüe R (2012). Differences in impact of patient and prosthetic characteristics on oral healthrelated quality of life among implant-retained overdenture wearers. J Dent.

[6] Slade GD, Spencer AJ (1994). Development and evaluation of the oral health impact profile. Community Dent Health.

[7] Locker D (1988). Measuring oral health: a conceptual framework. Community Dent Health.

[8] Awad M, Al-Shamrany M, Locker D, Allen F, Feine J (2008). Effect of reducing the number of items of the oral health impact profile on responsiveness, validity and reliability in edentulous populations. Community Dent Oral Epidemiol.

[9] Hebling E, Pereira AC (2007). Oral health-related quality of life: a critical appraisal of assessment tools used in elderly people. Gerodontology.

[10] Slade GD (1997). Derivation and validation of a short-form oral health impact profile. Community Dent Oral Epidemiol.

[11] Guillemin F, Bombardier C, Beaton D (1993 ). Cross-cultural adaptation of health-related quality of life measures: literature review and proposed guidelines. J Clin Epidemiol.

[12] Montero-Martín J, Bravo-Pérez M, Albaladejo-Martínez A, Hernández-Martín LA, Rosel-Gallardo EM (2009). Validation the oral health impact profile (OHIP-14sp) for adults in Spain. Med Oral Patol Oral Cir Bucal.

[13] Sonis ST (1998 ). Mucositis as a biological process: a new hypothesis for the development of chemotherapy-induced stomatotoxicity. Oral Oncol.

[14] Montero J, López JF, Galindo MP, Vicente P, Bravo M (2009). Impact of prosthodontic status on oral wellbeing: a cross-sectional cohort study. J Oral Rehabil.

[15] Montero J, López JF, Vicente MP, Galindo MP, Albaladejo A, Bravo M (2011). Comparative validity of the OIDP and OHIP-14 in describing the impact of oral health on quality of life in a cross-sectional study performed in Spanish adults. Med Oral Patol Oral Cir Bucal.

[16] Awad MA, Locker D, Korner-Bitensky N, Feine JS (2000). Measuring the effect of intra-oral implant rehabilitation on health-related quality of life in a randomized controlled clinical trial. J Dent Res.

[17] Montero J, Yarte JM, Bravo M, López-Valverde A (2011). Oral health-related quality of life of a consecutive sample of Spanish dental patients. Med Oral Patol Oral Cir Bucal.

[18] Souza RF, Patrocínio L, Pero AC, Marra J, Compagnoni MA (2007). Reliability and validation of a Brazilian version of the Oral Health Impact Profile for assessing edentulous subjects. J Oral Rehabil.

[19] John MT, Patrick DL, Slade GD (2002). The German version of the Oral Health Impact Profile--translation and psychometric properties. Eur J Oral Sci.

[20] Emami E, Heydecke G, Rompré PH, de Grandmont P, Feine JS (2009). Impact of implant support for mandibular dentures on satisfaction, oral and general health-related quality of life: a meta-analysis of randomized-controlled trials. Clin Oral Implants Res.

[21] de Oliveira TR, Frigerio ML (2004 ). Association between nutrition and the prosthetic condition in edentulous elderly. Gerodontology.

[22] Bucca C, Carossa S, Pivetti S, Gai V, Rolla G, Preti G (1999 ). Edentulism and worsening of obstructive sleep apnoea. Lancet.

[23] McGrath C, Bedi R (1998). A study of the impact of oral health on the quality of life of older people in the UK--findings from a national survey. Gerodontology.

[24] Lin HC, Corbet EF, Lo EC (2001). Oral mucosal lesions in adult Chinese. J Dent Res.

[25] van Waas MA (1990 ). The influence of clinical variables on patients' satisfaction
with complete dentures. J Prosthet Dent.

[26] Fenlon MR, Sherriff M (2008). An investigation of factors influencing patients' satisfaction with new complete dentures using structural equation modelling. J Dent.

[27] Boerrigter EM, Geertman ME, Van Oort RP, Bouma J, Raghoebar GM, van Waas MA (1995 ). Patient satisfaction with implant-retained mandibular overdentures. A comparison with new complete dentures not retained by implants--a multicentre randomized clinical trial. Br J Oral Maxillofac Surg.

[28] Hogenius S, Berggren U, Blomberg S, Jemt T, Ohman SC (1992). Demographical, odontological, and psychological variables in individuals referred for osseointegrated dental implants. Community Dent Oral Epidemiol.

[29] Portenoy RK (2011 ). Treatment of cancer pain. Lancet.

[30] Michaud PL, de Grandmont P, Feine JS, Emami E (2012). Measuring patient-based outcomes: Is treatment satisfaction associated with oral health-related quality of life?. J Dent.

